# Concept mapping as a method to teach an evidence-based educated medical topic: a comparative study in medical students

**DOI:** 10.1186/s40200-014-0086-1

**Published:** 2014-11-01

**Authors:** Farzane Saeidifard, Kazem Heidari, Moein Foroughi, Akbar Soltani

**Affiliations:** Endocrinology and Metabolism Research Center, Endocrinology and Metabolism Clinical Sciences Institute, Tehran University of Medical Sciences, Tehran, Iran; Department of Epidemiology and Biostatistics, School of Public Health, Tehran University of Medical Sciences, Tehran, Iran; Chronic Respiratory Diseases Research Center, National Research Institute of Tuberculosis and Lung Diseases (NRITLD), Shahid Beheshti University of Medical Sciences, Tehran, Iran

**Keywords:** Medical education, Concept mapping, Evidence-based medicine, Lecture-based teaching, Medical students

## Abstract

**Background:**

The objective of this study was to compare concept mapping with lecture-based method in teaching of evidence based educated topic to medical students.

**Methods:**

This randomized controlled trial was carried out on medical students during sixth year of 7-year MD curriculum clerkship phase. Cluster randomization was used to divide students into intervention and control groups. Both groups, at the beginning, were taught “Diabetic Ketoacidosis” (DKA) using evidence-based tool named Critically Appraised Topics (CAT). Students of intervention group were taught construction of concept maps on DKA and in the control group students had a lecture and a group discussion about what they had been taught on DKA. In the end, all of the students had an exam that they had to answer to 7 questions following to two clinical scenarios. The questions addressed physiopathology, diagnosis and treatment of patients with DKA and were scored separately. Sum of these scores was considered as total score. Scores were compared between intervention and control groups.

**Results:**

Seventy six medical students (28 male, 48 female) were participated in this study. Total score among intervention group was higher than control group (78.2% vs. 72.5%, p < 0.001). Subgroup analysis revealed significant differences between scores of students in the intervention group and scores of students in the control group in the diagnostic section of questions (81.0% vs. 71.5%, P < 0.001). The scores of students in the intervention group were also significantly higher than control group in physiopathology section of questions. No statistically significant difference was discovered between two groups in scores of answers to treatment section of questions (78.1 (7.3) vs. 72.5 (5.5) P = 0.03).

**Conclusion:**

The results of the study showed that concept mapping method was more successful in education of evidence-based educated topic via CATs in comparison with lecture-based method. Interpretation of this finding would be the concept mapping method may develop meaningful learning among medical students.

## Introduction

Lecture based teaching method, which was used for several years, is doubtful to be efficient enough and memory retention by this method after 6 months is less than 5 percent [1]. So it seems that an alternative method needs replacing. Within recent years, there has been an increasing interest in concept mapping (CM) as a powerful tool to educate new knowledge. Meaningful learning theory by Ausubel mentioned that conceptual learning occurs by hierarchically locating concepts, differentiation of concepts in the cognitive structure and connection between different concepts [2]. Based on this theory concept maps were introduced as schematic devices for representing a set of concepts in a framework of propositions. Concept maps are used in different fields such as accessing representations, communication, co-operation, staff development and critical thinking. In the field of education, they have been used in teaching or learning of new concepts in undergraduate education, curriculum planning and assessment of education. CM helps students integrate their basic and clinical knowledge and realize new information that they learn [3]. It can also enhance students' reasoning ability and can be used as an alternative of traditional nursing care plans for clinical preparation of students [4].

The concept of evidence based medicine (EBM) is widely accepted in undergraduate medical education curriculum and across different specialties [5-9]. Evidence-based education requires ready access to modern information resources. Characteristics and level of audiences' knowledge, the level of familiarity with EBM principles and the available setting and time are the most major determinants of best approach to evidence-based teaching [10]. In the region of evidence based medical education Critically Appraised Topics (CATs) have been introduced as one of the most powerful tools [11]. Critically Appraised Topic (CAT) is a standardized, 1-2 page summary of evidences related to a clinical question. These tools critically review methodological quality of evidences and clinical relevance of them and help physicians with limited time or appraisal skill, easily access to scientific literature [12].

Although the positive effect of using of CATs in medical education has been discussed previously, it seems that the method of teaching of these tools is also important. This study indicates on teaching evidence-based educated topic among medical students. The Objective of this study was to compare two educational methods (CM and lecture-based methods) in education of an evidence-based educated medical topic via CATs among medical students.

## Methods

This randomized controlled trial was carried out on medical students during internal medicine practical course in Shariati hospital (Tehran University of medical sciences) in the spring and the summer of 2007 by accessible sampling. They were the sixth year medical students in the clerkship phase of 7-year MD curriculum. According to this curriculum students have to spend practical courses on different medical specialty to acquire the ability of managing patients. All of the students were 22 to 24 years old. They were attended to the study during their 2-week endocrinology practical course. Students groups (including 4 groups that each group included 6-8 students) were randomly divided into intervention and control groups by cluster randomization method.

All of the students had spent a course to learn the physiopathology of disease prior to their practical course. Scores of physiopathology course of endocrine diseases were also gained from medical faculty. Each group of the students was allocated to take intervention or control education.

All of the students were introduced to basic concepts of EBM including making a structured question based on PICO (P (patient) I (intervention) C (comparison) O (outcome)) and principles of critically appraise of diagnosis and treatment oriented studies through a 4-hour work shop (a separate workshop was held for each group during their practical course of endocrinology). Afterward three Critically Appraised Topics (CAT) were reviewed by an endocrinologist. [S.A] that were taken from Best Evidence Topics (Best Bets 2006, http://www.bestbets.org). The titles of the CATs were the followings:

1. In [an adult patient with diabetic ketoacidosis] do [venous blood gases] accurately demonstrate [the degree of acidosis]?
2. In [diabetic ketoacidosis], does [an initial bolus of insulin result in faster, safer restoration of normoglycaemia and pH] when compared [with no bolus prior to commencing continuous insulin infusion]?
3. In [adults with diabetic ketoacidosis and significant acidaemia] is [the additional administration of iv bicarbonate better than standard fluid resuscitation] give [more rapid improvement of biochemical parameters and an increased survival]

At the beginning of the study all of students were asked for their familiarity with CAT. None of them were familiar with it. The endocrinologist who was also expert in EBM taught DKA to the students using these CATs. The first CAT addressed physiopathology and diagnosis of DKA and the second and the third ones included some therapeutic information for the students.

### Intervention groups

In these groups, after the preceding stage, students were introduced to CM principles in a 45-minute session (none of them were familiar with CM method). Topics which were considered in this stage included meaningful learning, concept map introduction and the instruction of constructing, assessing and scoring of concept maps. Afterward each group drew a concept map by a method known as “selected term CM”. Students drew concept maps in diabetic ketoacidosis using key concepts that were introduced to them. Key concepts included these words: DKA, diagnosis, treatment, blood sugar, insulin, bicarbonate, ketonemia, ketonuria, acidosis, blood gas, potassium, sodium, normal saline, hyperkalemia, hypokalemia, hyponatremia, etiology, and pancreas. A facilitator accompanied them in the process of drawing maps. After drawing the concept maps, students discussed about the maps with the facilitator and the endocrinologist.

### Control groups

Students who had been assigned to these groups after the first step of becoming familiar with EBM and CAT, had a 45-minute session of a lecture and a group discussion about DKA. No facilitator guided their discussion but an equal timeframe was allocated to each student to discuss.

### Assessment

After the educational session, all of the students in each group were attended in an exam including two clinical scenarios of patients suspected of DKA (Appendix 1) followed by 4 and 3 open-answer questions (respectively) on physiopathology, diagnosis and treatment of patients that had been presented in scenarios. The exam administered immediately after the educational session. A pre-prepared answer sheet, which was conducted via an expert panel, was used for scoring of the answers. Each part of the questions (physiopathology, diagnosis and treatment parts) was scored separately. Sum of these scores was considered as total score. Scores were compared between intervention and control groups using Mann-Whitney test. Correlation between total scores and also scores of different parts with scores of physiopathology course of endocrinology were assessed using standard correlation tests. All of the statistical analysis was performed using SPSS ver.13. Significant level was considered as p-value ≤0.05.

The ethics committee of Tehran University of Medical Sciences had approved the protocol of this study and the study was eligible for a waiver of written informed consent.

## Results

Seventy six medical students (28 male (M), 48 female (F)) were participated in this study. After randomization of groups, 37 students (14 M, 23 F) were allocated to the intervention groups and 39 out of them (14 M, 25 F) to the control groups. There was not any refusal and all of the students completed the course.

The mean score for physiopathology course of endocrinology was 15.15 out of 20 (SD = 1.87) in the intervention groups and 15.50 out of 20 (SD = 1.51) in the control groups. There was not statistically significant difference between these scores.

Evaluation of post education exam scores between these two groups showed that students in the intervention groups had significantly better scores compared to the control groups (78.2% vs. 72.5% of total score, P < 0.001). About the diagnostic section of questions students in the intervention groups had answered significantly better than control groups. (81.0% vs. 71.5%, P < 0.001) (Table 1). The score of physiopathology section of questions among intervention groups was also significantly better than control groups. (78.1 (7.3) vs. 72.5 (5.5) P = 0.03). There was no statistical significant difference in the scores of answers to treatment section of questions between intervention and control groups.

**Table 1:** **Comparison of medical students’ characteristics and final exam scores in two groups of control and intervention**

	**Intervention group (n = 37)**	**Control group**	**P value**
Sex			0.86*
**Male (%)**	14 (37.8)	14 (35.9)	
**Female (%)**	23 (62.2)	25 (64.1)	
**Physiopathology course score (out of 20)**	15.15 (1.87)	15.54 (1.51)	0.313**
**Physiopathology section score (%)**	78.1 (7.3)	72.5 (5.5)	0.03**
**Diagnosis section score (%)**	81.08 (11.2)	71.53 (8.1)	<0.001**
**Treatment section score (%)**	75.54 (10.5)	71.47 (7.8)	0.07**
**Total score (%)**	78.2 (7.85)	72.5 (5.5)	<0.001**

*: Chi^2^ test.

**: Mann-Whitney U test.

Table 2 displays relation between scores of physiopathology course of endocrinology and student’s exam score.

**Table 2:** **Correlation between non clinical pathophysiology course score of medical students and final exam scores**

	**Intervention group (n = 37)**	**Control group (n = 39)**	**Total (n = 76)**
**Pathophysiology section score (%)**	0.02	-0.16	-0.85
**Diagnosis section score (%)**	-0.007	-0.19	-0.12
**Treatment section score (%)**	-0.11	0.36*	-0.23*
**Total score (%)**	-0.04	-0.33	-0.18

*: P < 0.05.

## Discussion

In this study we compared two educational methods in education of evidence-based educated topic. The results show that CM method works better in comparison with lecture-based method in education of an evidence-based educated medical topic via CATs. We found that the mean score of students in the intervention groups was significantly higher than the control groups (78.2% vs. 72.5% of total score, P<0.001). Previous studies indicate the positive role of CM method in medical education curriculum by EBM assistance [13-17]. CATs as tools for evidence-based learning can stimulate learning of new topics and so its teaching method would be important. However in our study both groups used this tool, students of intervention groups learned better. These findings show the positive role of CM on learning of medical topic via CATs.

The vast majority of studies have indicated on the role of CM in development of meaningful learning and problem solving [18-20]. They indicate that through concept mapping students could integrate basic and clinical knowledge and move from linear thinking patterns to more integrated holistic patterns. In some studies integration of CM with problem-based learning (PBL) has been indicated. They indicated that concept mapping stimulated meaningful learning within a PBL course [21,22].

The discrepancy between our study and above-mentioned ones is in indication of use of EBM-related tools in medical education. However in those studies using of concept maps has been mentioned as effective tools, importance of EBM has been neglected in medical education. In present study indication was on the integration of EBM and CM in education of medical topics. Since within recent decades EBM has introduced as a new and efficient way in gathering and using of the best and the most relevant evidences in practice, the methods of education of its tools would be important.

Surprisingly we found that in subgroup analysis of the answers students in the intervention groups had significantly better scores in physiopathology and diagnostic sections of questions while the difference between their scores in treatment section were statistically non-significant. As an explanation for this finding it can be mention that prior to entrance to the practical course, students receive trainings on physiopathology basis of diseases as physiopathology course. Physiopathology and feature of diseases get the most importance and attention in the curriculum of medical education in Iran while the treatment of patients is of the secondary importance. The physiopathology and diagnostic sections of present study were a redundancy of students’ prior knowledge in these areas while the treatment section was somewhat new.

Several limitations of this study deserve comment. Only one rater performed scoring of the exam. This factor can reduce reliability of scores. In the control group, we didn’t have a facilitator as same as the intervention group. Facilitator helped students draw maps and also motivated them to learn. The second mentioned role of the facilitator might affect our result. The similarity of knowledge of students before the educational intervention was not assessed. The difference between their prior knowledge could cause bias. The final exam was performed immediately after the education session. This exam couldn’t assess students’ recall of knowledge, but it can be concluded that by CM method, students learn and process better and more rapid. By this design, maturation bias was diminished. Although students were requested to conceal the details of the study from other students and due to presence of students in different wards at the same time contamination risk was low, it was not zero. This probability of contamination could affect the process and the result of the study.

## Conclusion

In this study we found that CM as educational method was better than lecture-based traditional method on education of an evidence-based educated medical topic via CATs. In conclusion, in the field of medical education and especially in the field of EBM, simplicity of learning by CM is an opportunity which may help medical students learn and criticize better many more concepts and evidences which they encountered with them. So they will be able to use and understand faster the best and the most relevant evidences to judge better about their patients. This might ultimate in better treatment.

## Appendix 1

### Case 1

A 21 year old man developed polyuria, polydipsia and blurred vision. On examination he was noted to have persistent blood pressure reading of 100/60 mmhg, a low grade fever (38.5°C), heart rate of 110 bpm and respiratory rate of 14 breaths per minute. Head and neck exam reveals poor dentition with significant periodontal disease. Chest and cardiac exam were normal. Abdominal exam was remarkable for central obesity. The extremities showed normal sensation and vibratory sense with a 10 gram monofilament. Laboratory examination was notable for a white cell count of 17000, blood glucose of 480 mg/dl, an anion gap of 27, with a pH of 7.10 and serum ketones positive at 1:16.

Questions:

1. Describe the major causative factors of DKA.
2. List the clinical sign and symptoms of diabetes and DKA.
3. Describe the major metabolic change in DKA.
4. Discuss the major objectives of medical management of DKA.

### Case 2

A 22 year old medical student admitted by confusion and agitation. He has had nausea, vomiting and abdominal pain for 2 last days and polyuria, polydipsia and nocturia since 2 weeks ago. On physical examination he had moderate xerostomy with normal chest, abdominal and extremities exam. Laboratory findings were as following:

BS = 458 WBC = 14500 U/A: keton bodies = +++ glu = +++

Na = 129 PMN = 85% ABG: PH = 7.1 PCO_2_ = 88 mmHg PO_2_ = 88 mmHg

K = 4.9 lymph = 12% HCO_3_ = 5 mmol/lit

Cr = 1.8 mono = 2%

Baso = 1%

Questions:

1. Prescription of insulin bolus in DKA, is it necessary?
2. Use of bicarbonate in adult with diabetic ketoacidosis, is it necessary?
3. Venous blood gas in adult patients with diabetic ketoacidosis, is it necessary?

## Abbreviations

DKA: Diabetic ketoacidosis
EBM: Evidence-Based medicine
CAT: Critically appraised topic
CM: Concept mapping
PICO: Patient, intervention, comparison, outcome
SD: Standard deviation
